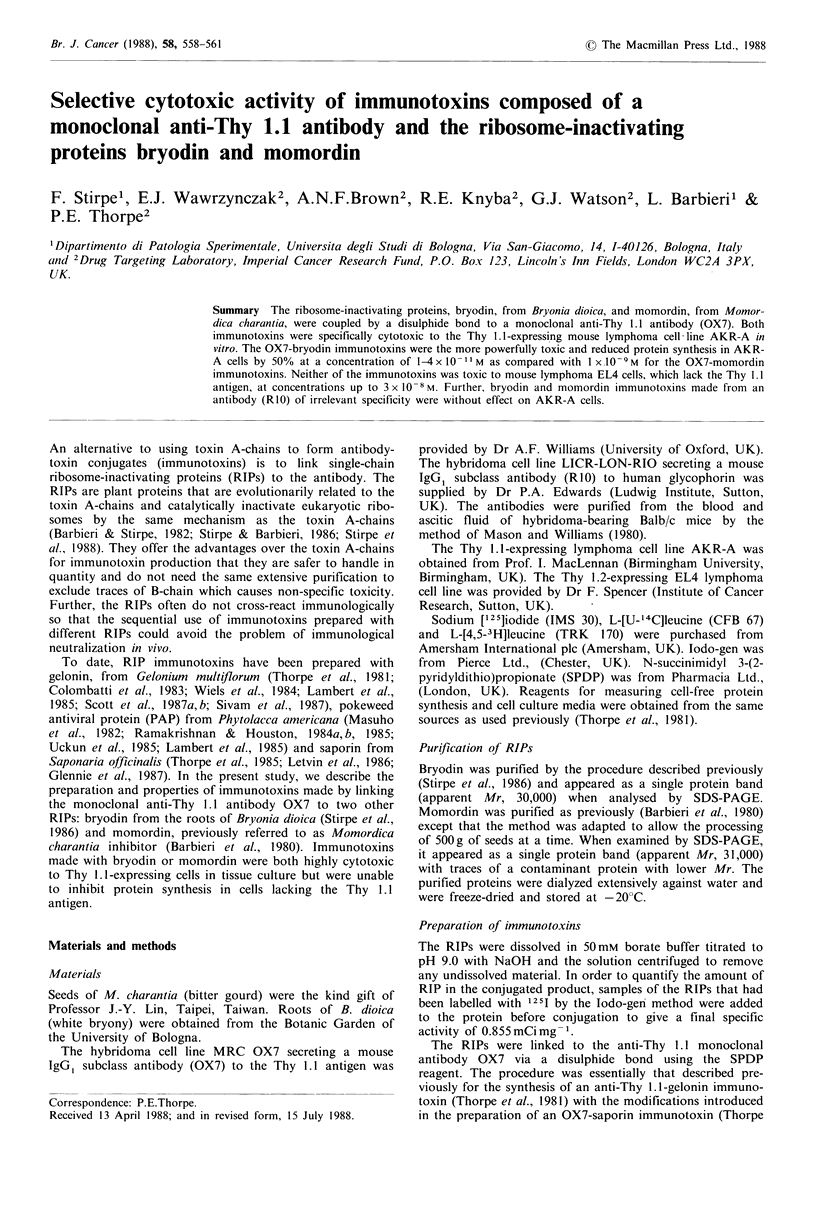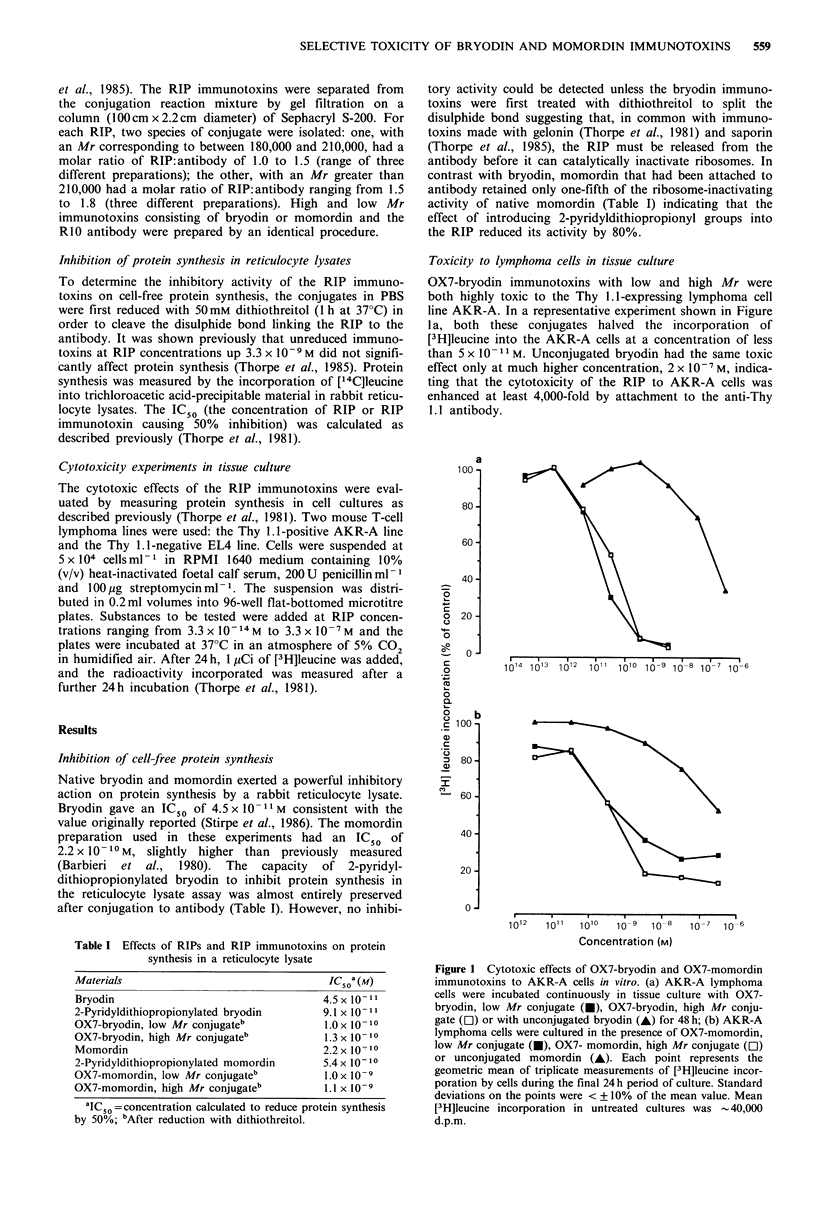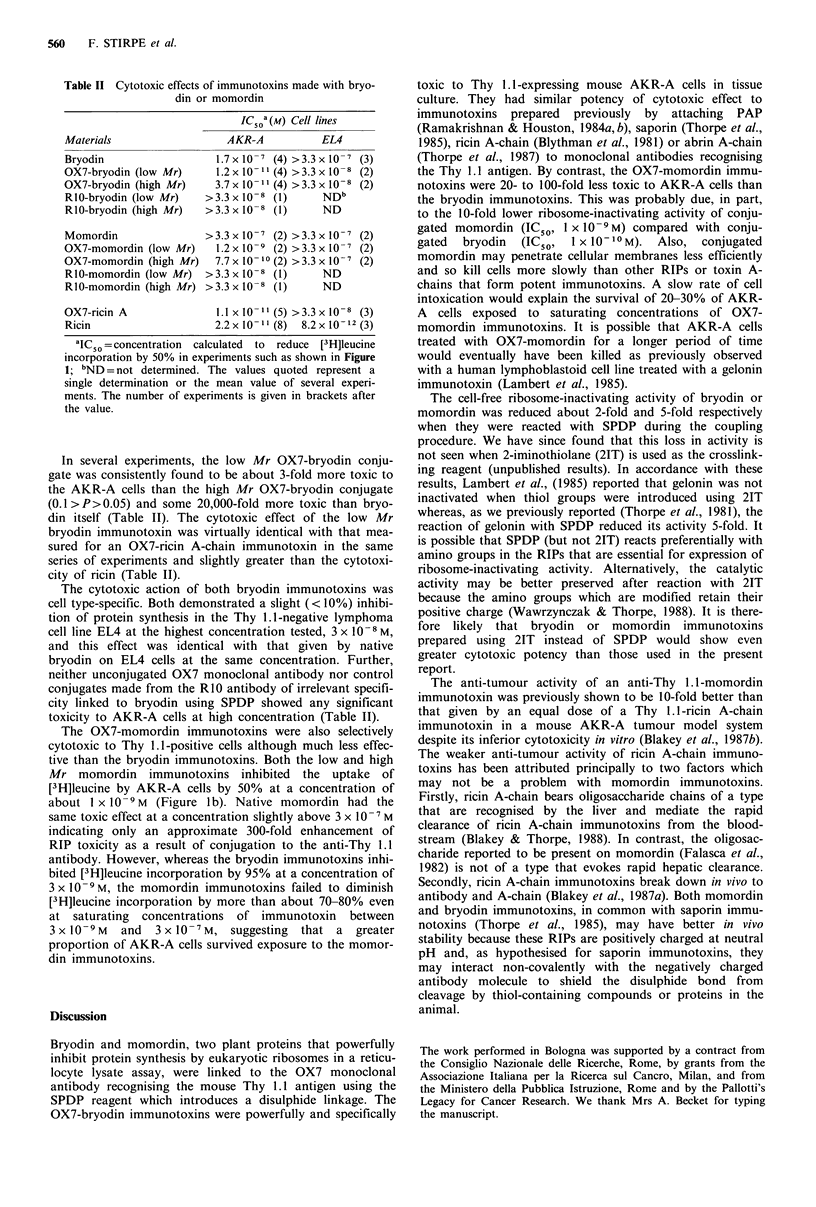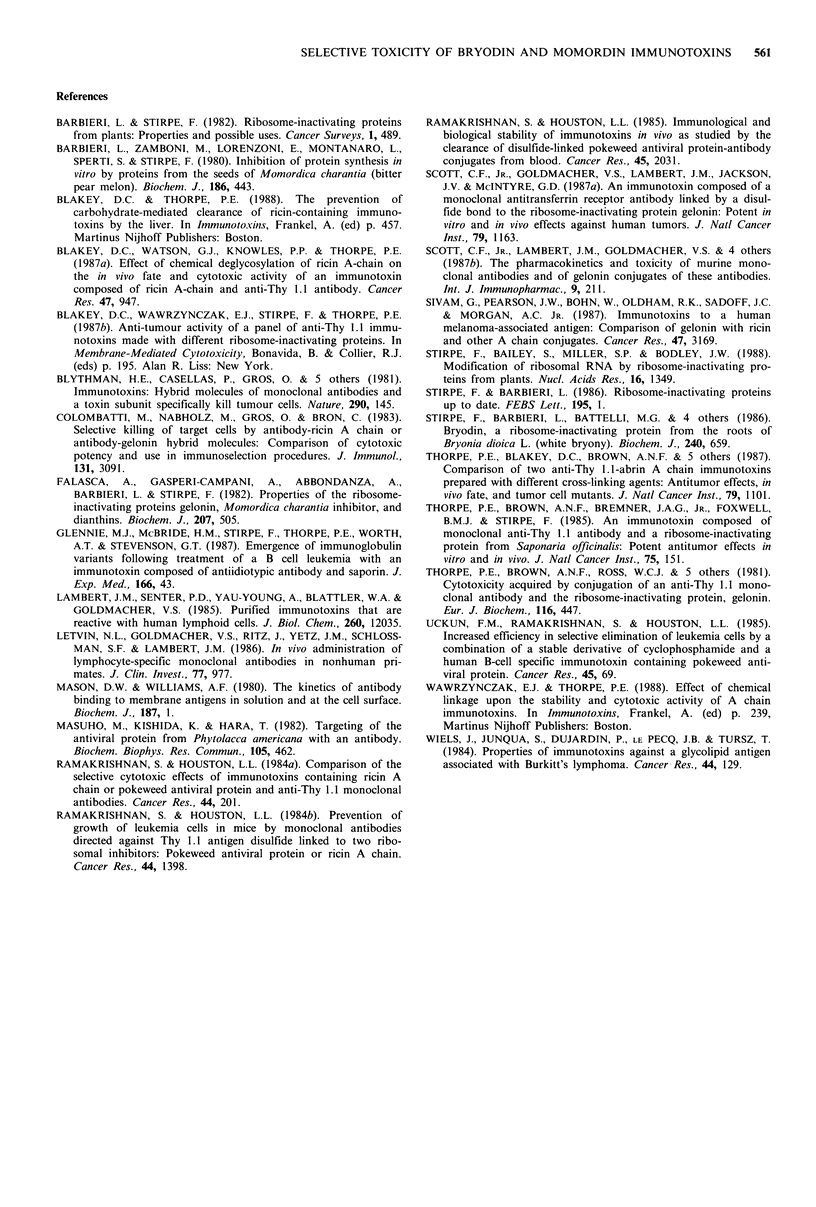# Selective cytotoxic activity of immunotoxins composed of a monoclonal anti-Thy 1.1 antibody and the ribosome-inactivating proteins bryodin and momordin.

**DOI:** 10.1038/bjc.1988.258

**Published:** 1988-11

**Authors:** F. Stirpe, E. J. Wawrzynczak, A. N. Brown, R. E. Knyba, G. J. Watson, L. Barbieri, P. E. Thorpe

**Affiliations:** Dipartimento di Patologia Sperimentale, Universita degli Studi di Bologna, Italy.

## Abstract

The ribosome-inactivating proteins, bryodin, from Bryonia dioica, and momordin, from Momordica charantia, were coupled by a disulphide bond to a monoclonal anti-Thy 1.1 antibody (OX7). Both immunotoxins were specifically cytotoxic to the Thy 1.1-expressing mouse lymphoma cell line AKR-A in vitro. The OX7-bryodin immunotoxins were the more powerfully toxic and reduced protein synthesis in AKR-A cells by 50% at a concentration of 1-4 x 10(-11) M as compared with 1 x 10(-9) M for the OX7-momordin immunotoxins. Neither of the immunotoxins was toxic to mouse lymphoma EL4 cells, which lack the Thy 1.1 antigen, at concentrations up to 3 x 10(-8) M. Further, bryodin and momordin immunotoxins made from an antibody (R10) of irrelevant specificity were without effect on AKR-A cells.


*Br. J. Cancer* (1988), **58**, 558–561 © The Macmillan Press Ltd., 1988

### Selective cytotoxic activity of immunotoxins composed of a monoclonal anti-Thy 1.1 antibody and the ribosome-inactivating proteins bryodin and momordin

F. Stirpe[1], E.J. Wawrzynczak[2], A.N.F.Brown[2], R.E. Knyba[2], G.J. Watson[2], L. Barbieri[1] & P.E. Thorpe[2]

[1]*Dipartimento di Patologia Sperimentale, Universita degli Studi di Bologna, Via San-Giacomo, 14, I-40126, Bologna, Italy and* [2]*Drug Targeting Laboratory, Imperial Cancer Research Fund, P.O. Box 123, Lincoln's Inn Fields, London WC2A 3PX, UK.*

**Summary** The ribosome-inactivating proteins, bryodin, from *Bryonia dioica*, and momordin, from *Momordica charantia*, were coupled by a disulphide bond to a monoclonal anti-Thy 1.1 antibody (OX7). Both immunotoxins were specifically cytotoxic to the Thy 1.1-expressing mouse lymphoma cell line AKR-A *in vitro*. The OX7-bryodin immunotoxins were the more powerfully toxic and reduced protein synthesis in AKR-A cells by 50% at a concentration of $1–4 \times 10^{-11}$ M as compared with $1 \times 10^{-9}$ M for the OX7-momordin immunotoxins. Neither of the immunotoxins was toxic to mouse lymphoma EL4 cells, which lack the Thy 1.1 antigen, at concentrations up to $3 \times 10^{-8}$ M. Further, bryodin and momordin immunotoxins made from an antibody (R10) of irrelevant specificity were without effect on AKR-A cells.

An alternative to using toxin A-chains to form antibody-toxin conjugates (immunotoxins) is to link single-chain ribosome-inactivating proteins (RIPs) to the antibody. The RIPs are plant proteins that are evolutionarily related to the toxin A-chains and catalytically inactivate eukaryotic ribosomes by the same mechanism as the toxin A-chains (Barbieri & Stirpe, 1982; Stirpe & Barbieri, 1986; Stirpe *et al.*, 1988). They offer the advantages over the toxin A-chains for immunotoxin production that they are safer to handle in quantity and do not need the same extensive purification to exclude traces of B-chain which causes non-specific toxicity. Further, the RIPs often do not cross-react immunologically so that the sequential use of immunotoxins prepared with different RIPs could avoid the problem of immunological neutralization *in vivo*.

To date, RIP immunotoxins have been prepared with gelonin, from *Gelonium multiflorum* (Thorpe *et al.*, 1981; Colombatti *et al.*, 1983; Wiels *et al.*, 1984; Lambert *et al.*, 1985; Scott *et al.*, 1987*a,b*; Sivam *et al.*, 1987), pokeweed antiviral protein (PAP) from *Phytolacca americana* (Masuho *et al.*, 1982; Ramakrishnan & Houston, 1984*a,b*, 1985; Uckun *et al.*, 1985; Lambert *et al.*, 1985) and saporin from *Saponaria officinalis* (Thorpe *et al.*, 1985; Letvin *et al.*, 1986; Glennie *et al.*, 1987). In the present study, we describe the preparation and properties of immunotoxins made by linking the monoclonal anti-Thy 1.1 antibody OX7 to two other RIPs: bryodin from the roots of *Bryonia dioica* (Stirpe *et al.*, 1986) and momordin, previously referred to as *Momordica charantia* inhibitor (Barbieri *et al.*, 1980). Immunotoxins made with bryodin or momordin were both highly cytotoxic to Thy 1.1-expressing cells in tissue culture but were unable to inhibit protein synthesis in cells lacking the Thy 1.1 antigen.

### Materials and methods

### *Materials*

Seeds of *M. charantia* (bitter gourd) were the kind gift of Professor J.-Y. Lin, Taipei, Taiwan. Roots of *B. dioica* (white bryony) were obtained from the Botanic Garden of the University of Bologna.

The hybridoma cell line MRC OX7 secreting a mouse $IgG_1$ subclass antibody (OX7) to the Thy 1.1 antigen was provided by Dr A.F. Williams (University of Oxford, UK). The hybridoma cell line LICR-LON-RIO secreting a mouse $IgG_1$ subclass antibody (R10) to human glycophorin was supplied by Dr P.A. Edwards (Ludwig Institute, Sutton, UK). The antibodies were purified from the blood and ascitic fluid of hybridoma-bearing Balb/c mice by the method of Mason and Williams (1980).

The Thy 1.1-expressing lymphoma cell line AKR-A was obtained from Prof. I. MacLennan (Birmingham University, Birmingham, UK). The Thy 1.2-expressing EL4 lymphoma cell line was provided by Dr F. Spencer (Institute of Cancer Research, Sutton, UK).

Sodium [$^{125}$I]iodide (IMS 30), L-[U-$^{14}$C]leucine (CFB 67) and L-[4,5-$^{3}$H]leucine (TRK 170) were purchased from Amersham International plc (Amersham, UK). Iodo-gen was from Pierce Ltd., (Chester, UK). N-succinimidyl 3-(2-pyridyldithio)propionate (SPDP) was from Pharmacia Ltd., (London, UK). Reagents for measuring cell-free protein synthesis and cell culture media were obtained from the same sources as used previously (Thorpe *et al.*, 1981).

### *Purification of RIPs*

Bryodin was purified by the procedure described previously (Stirpe *et al.*, 1986) and appeared as a single protein band (apparent *Mr*, 30,000) when analysed by SDS-PAGE. Momordin was purified as previously (Barbieri *et al.*, 1980) except that the method was adapted to allow the processing of 500 g of seeds at a time. When examined by SDS-PAGE, it appeared as a single protein band (apparent *Mr*, 31,000) with traces of a contaminant protein with lower *Mr*. The purified proteins were dialyzed extensively against water and were freeze-dried and stored at −20°C.

### *Preparation of immunotoxins*

The RIPs were dissolved in 50 mM borate buffer titrated to pH 9.0 with NaOH and the solution centrifuged to remove any undissolved material. In order to quantify the amount of RIP in the conjugated product, samples of the RIPs that had been labelled with $^{125}$I by the Iodo-gen method were added to the protein before conjugation to give a final specific activity of 0.855 mCi $mg^{-1}$.

The RIPs were linked to the anti-Thy 1.1 monoclonal antibody OX7 via a disulphide bond using the SPDP reagent. The procedure was essentially that described previously for the synthesis of an anti-Thy 1.1-gelonin immunotoxin (Thorpe *et al.*, 1981) with the modifications introduced in the preparation of an OX7-saporin immunotoxin (Thorpe

Correspondence: P.E.Thorpe.
Received 13 April 1988; and in revised form, 15 July 1988.

*et al.*, 1985). The RIP immunotoxins were separated from the conjugation reaction mixture by gel filtration on a column (100 cm x 2.2 cm diameter) of Sephacryl S-200. For each RIP, two species of conjugate were isolated: one, with an *Mr* corresponding to between 180,000 and 210,000, had a molar ratio of RIP:antibody of 1.0 to 1.5 (range of three different preparations); the other, with an *Mr* greater than 210,000 had a molar ratio of RIP:antibody ranging from 1.5 to 1.8 (three different preparations). High and low *Mr* immunotoxins consisting of bryodin or momordin and the R10 antibody were prepared by an identical procedure.

### *Inhibition of protein synthesis in reticulocyte lysates*

To determine the inhibitory activity of the RIP immunotoxins on cell-free protein synthesis, the conjugates in PBS were first reduced with 50 mM dithiothreitol (1 h at 37°C) in order to cleave the disulphide bond linking the RIP to the antibody. It was shown previously that unreduced immunotoxins at RIP concentrations up $3.3 \times 10^{-9}$ M did not significantly affect protein synthesis (Thorpe *et al.*, 1985). Protein synthesis was measured by the incorporation of [$^{14}$C]leucine into trichloroacetic acid-precipitable material in rabbit reticulocyte lysates. The $IC_{50}$ (the concentration of RIP or RIP immunotoxin causing 50% inhibition) was calculated as described previously (Thorpe *et al.*, 1981).

### *Cytotoxicity experiments in tissue culture*

The cytotoxic effects of the RIP immunotoxins were evaluated by measuring protein synthesis in cell cultures as described previously (Thorpe *et al.*, 1981). Two mouse T-cell lymphoma lines were used: the Thy 1.1-positive AKR-A line and the Thy 1.1-negative EL4 line. Cells were suspended at $5 \times 10^4$ cells ml$^{-1}$ in RPMI 1640 medium containing 10% (v/v) heat-inactivated foetal calf serum, 200 U penicillin ml$^{-1}$ and 100 $\mu$g streptomycin ml$^{-1}$. The suspension was distributed in 0.2 ml volumes into 96-well flat-bottomed microtitre plates. Substances to be tested were added at RIP concentrations ranging from $3.3 \times 10^{-14}$ M to $3.3 \times 10^{-7}$ M and the plates were incubated at 37°C in an atmosphere of 5% $CO_2$ in humidified air. After 24 h, 1 $\mu$Ci of [$^3$H]leucine was added, and the radioactivity incorporated was measured after a further 24 h incubation (Thorpe *et al.*, 1981).

### Results

### *Inhibition of cell-free protein synthesis*

Native bryodin and momordin exerted a powerful inhibitory action on protein synthesis by a rabbit reticulocyte lysate. Bryodin gave an $IC_{50}$ of $4.5 \times 10^{-11}$ M consistent with the value originally reported (Stirpe *et al.*, 1986). The momordin preparation used in these experiments had an $IC_{50}$ of $2.2 \times 10^{-10}$ M, slightly higher than previously measured (Barbieri *et al.*, 1980). The capacity of 2-pyridyldithiopropionylated bryodin to inhibit protein synthesis in the reticulocyte lysate assay was almost entirely preserved after conjugation to antibody (Table I). However, no inhibitory activity could be detected unless the bryodin immunotoxins were first treated with dithiothreitol to split the disulphide bond suggesting that, in common with immunotoxins made with gelonin (Thorpe *et al.*, 1981) and saporin (Thorpe *et al.*, 1985), the RIP must be released from the antibody before it can catalytically inactivate ribosomes. In contrast with bryodin, momordin that had been attached to antibody retained only one-fifth of the ribosome-inactivating activity of native momordin (Table I) indicating that the effect of introducing 2-pyridyldithiopropionyl groups into the RIP reduced its activity by 80%.

**Table I** Effects of RIPs and RIP immunotoxins on protein synthesis in a reticulocyte lysate

| *Materials* | $IC_{50}$[a] (*M*) |
|---|---|
| Bryodin | $4.5 \times 10^{-11}$ |
| 2-Pyridyldithiopropionylated bryodin | $9.1 \times 10^{-11}$ |
| OX7-bryodin, low *Mr* conjugate[b] | $1.0 \times 10^{-10}$ |
| OX7-bryodin, high *Mr* conjugate[b] | $1.3 \times 10^{-10}$ |
| Momordin | $2.2 \times 10^{-10}$ |
| 2-Pyridyldithiopropionylated momordin | $5.4 \times 10^{-10}$ |
| OX7-momordin, low *Mr* conjugate[b] | $1.0 \times 10^{-9}$ |
| OX7-momordin, high *Mr* conjugate[b] | $1.1 \times 10^{-9}$ |

[a]$IC_{50}$ = concentration calculated to reduce protein synthesis by 50%; [b]After reduction with dithiothreitol.

### *Toxicity to lymphoma cells in tissue culture*

OX7-bryodin immunotoxins with low and high *Mr* were both highly toxic to the Thy 1.1-expressing lymphoma cell line AKR-A. In a representative experiment shown in Figure 1a, both these conjugates halved the incorporation of [$^3$H]leucine into the AKR-A cells at a concentration of less than $5 \times 10^{-11}$ M. Unconjugated bryodin had the same toxic effect only at much higher concentration, $2 \times 10^{-7}$ M, indicating that the cytotoxicity of the RIP to AKR-A cells was enhanced at least 4,000-fold by attachment to the anti-Thy 1.1 antibody.

**Figure 1** Cytotoxic effects of OX7-bryodin and OX7-momordin immunotoxins to AKR-A cells *in vitro*. (a) AKR-A lymphoma cells were incubated continuously in tissue culture with OX7-bryodin, low *Mr* conjugate (■), OX7-bryodin, high *Mr* conjugate (□) or with unconjugated bryodin (▲) for 48 h; (b) AKR-A lymphoma cells were cultured in the presence of OX7-momordin, low *Mr* conjugate (■), OX7- momordin, high *Mr* conjugate (□) or unconjugated momordin (▲). Each point represents the geometric mean of triplicate measurements of [$^3$H]leucine incorporation by cells during the final 24 h period of culture. Standard deviations on the points were $< \pm 10\%$ of the mean value. Mean [$^3$H]leucine incorporation in untreated cultures was ~40,000 d.p.m.

**Table II** Cytotoxic effects of immunotoxins made with bryodin or momordin

| | $IC_{50}$[a] (M) Cell lines | |
|---|---|---|
| *Materials* | *AKR-A* | *EL4* |
| Bryodin | $1.7\times10^{-7}$ (4) | $>3.3\times10^{-7}$ (3) |
| OX7-bryodin (low *Mr*) | $1.2\times10^{-11}$ (4) | $>3.3\times10^{-8}$ (2) |
| OX7-bryodin (high *Mr*) | $3.7\times10^{-11}$ (4) | $>3.3\times10^{-8}$ (2) |
| R10-bryodin (low *Mr*) | $>3.3\times10^{-8}$ (1) | ND[b] |
| R10-bryodin (high *Mr*) | $>3.3\times10^{-8}$ (1) | ND |
| Momordin | $>3.3\times10^{-7}$ (2) | $>3.3\times10^{-7}$ (2) |
| OX7-momordin (low *Mr*) | $1.2\times10^{-9}$ (2) | $>3.3\times10^{-7}$ (2) |
| OX7-momordin (high *Mr*) | $7.7\times10^{-10}$ (2) | $>3.3\times10^{-7}$ (2) |
| R10-momordin (low *Mr*) | $>3.3\times10^{-8}$ (1) | ND |
| R10-momordin (high *Mr*) | $>3.3\times10^{-8}$ (1) | ND |
| OX7-ricin A | $1.1\times10^{-11}$ (5) | $>3.3\times10^{-8}$ (3) |
| Ricin | $2.2\times10^{-11}$ (8) | $8.2\times10^{-12}$ (3) |

[a]$IC_{50}$ = concentration calculated to reduce [$^3$H]leucine incorporation by 50% in experiments such as shown in **Figure 1**; [b]ND = not determined. The values quoted represent a single determination or the mean value of several experiments. The number of experiments is given in brackets after the value.

In several experiments, the low *Mr* OX7-bryodin conjugate was consistently found to be about 3-fold more toxic to the AKR-A cells than the high *Mr* OX7-bryodin conjugate ($0.1 > P > 0.05$) and some 20,000-fold more toxic than bryodin itself (Table II). The cytotoxic effect of the low *Mr* bryodin immunotoxin was virtually identical with that measured for an OX7-ricin A-chain immunotoxin in the same series of experiments and slightly greater than the cytotoxicity of ricin (Table II).

The cytotoxic action of both bryodin immunotoxins was cell type-specific. Both demonstrated a slight (< 10%) inhibition of protein synthesis in the Thy 1.1-negative lymphoma cell line EL4 at the highest concentration tested, $3\times10^{-8}$ M, and this effect was identical with that given by native bryodin on EL4 cells at the same concentration. Further, neither unconjugated OX7 monoclonal antibody nor control conjugates made from the R10 antibody of irrelevant specificity linked to bryodin using SPDP showed any significant toxicity to AKR-A cells at high concentration (Table II).

The OX7-momordin immunotoxins were also selectively cytotoxic to Thy 1.1-positive cells although much less effective than the bryodin immunotoxins. Both the low and high *Mr* momordin immunotoxins inhibited the uptake of [$^3$H]leucine by AKR-A cells by 50% at a concentration of about $1\times10^{-9}$ M (Figure 1b). Native momordin had the same toxic effect at a concentration slightly above $3\times10^{-7}$ M indicating only an approximate 300-fold enhancement of RIP toxicity as a result of conjugation to the anti-Thy 1.1 antibody. However, whereas the bryodin immunotoxins inhibited [$^3$H]leucine incorporation by 95% at a concentration of $3\times10^{-9}$ M, the momordin immunotoxins failed to diminish [$^3$H]leucine incorporation by more than about 70–80% even at saturating concentrations of immunotoxin between $3\times10^{-9}$ M and $3\times10^{-7}$ M, suggesting that a greater proportion of AKR-A cells survived exposure to the momordin immunotoxins.

### Discussion

Bryodin and momordin, two plant proteins that powerfully inhibit protein synthesis by eukaryotic ribosomes in a reticulocyte lysate assay, were linked to the OX7 monoclonal antibody recognising the mouse Thy 1.1 antigen using the SPDP reagent which introduces a disulphide linkage. The OX7-bryodin immunotoxins were powerfully and specifically toxic to Thy 1.1-expressing mouse AKR-A cells in tissue culture. They had similar potency of cytotoxic effect to immunotoxins prepared previously by attaching PAP (Ramakrishnan & Houston, 1984*a*,*b*), saporin (Thorpe *et al.*, 1985), ricin A-chain (Blythman *et al.*, 1981) or abrin A-chain (Thorpe *et al.*, 1987) to monoclonal antibodies recognising the Thy 1.1 antigen. By contrast, the OX7-momordin immunotoxins were 20- to 100-fold less toxic to AKR-A cells than the bryodin immunotoxins. This was probably due, in part, to the 10-fold lower ribosome-inactivating activity of conjugated momordin ($IC_{50}$, $1\times10^{-9}$ M) compared with conjugated bryodin ($IC_{50}$, $1\times10^{-10}$ M). Also, conjugated momordin may penetrate cellular membranes less efficiently and so kill cells more slowly than other RIPs or toxin A-chains that form potent immunotoxins. A slow rate of cell intoxication would explain the survival of 20–30% of AKR-A cells exposed to saturating concentrations of OX7-momordin immunotoxins. It is possible that AKR-A cells treated with OX7-momordin for a longer period of time would eventually have been killed as previously observed with a human lymphoblastoid cell line treated with a gelonin immunotoxin (Lambert *et al.*, 1985).

The cell-free ribosome-inactivating activity of bryodin or momordin was reduced about 2-fold and 5-fold respectively when they were reacted with SPDP during the coupling procedure. We have since found that this loss in activity is not seen when 2-iminothiolane (2IT) is used as the crosslinking reagent (unpublished results). In accordance with these results, Lambert *et al.*, (1985) reported that gelonin was not inactivated when thiol groups were introduced using 2IT whereas, as we previously reported (Thorpe *et al.*, 1981), the reaction of gelonin with SPDP reduced its activity 5-fold. It is possible that SPDP (but not 2IT) reacts preferentially with amino groups in the RIPs that are essential for expression of ribosome-inactivating activity. Alternatively, the catalytic activity may be better preserved after reaction with 2IT because the amino groups which are modified retain their positive charge (Wawrzynczak & Thorpe, 1988). It is therefore likely that bryodin or momordin immunotoxins prepared using 2IT instead of SPDP would show even greater cytotoxic potency than those used in the present report.

The anti-tumour activity of an anti-Thy 1.1-momordin immunotoxin was previously shown to be 10-fold better than that given by an equal dose of a Thy 1.1-ricin A-chain immunotoxin in a mouse AKR-A tumour model system despite its inferior cytotoxicity *in vitro* (Blakey *et al.*, 1987*b*). The weaker anti-tumour activity of ricin A-chain immunotoxins has been attributed principally to two factors which may not be a problem with momordin immunotoxins. Firstly, ricin A-chain bears oligosaccharide chains of a type that are recognised by the liver and mediate the rapid clearance of ricin A-chain immunotoxins from the bloodstream (Blakey & Thorpe, 1988). In contrast, the oligosaccharide reported to be present on momordin (Falasca *et al.*, 1982) is not of a type that evokes rapid hepatic clearance. Secondly, ricin A-chain immunotoxins break down *in vivo* to antibody and A-chain (Blakey *et al.*, 1987*a*). Both momordin and bryodin immunotoxins, in common with saporin immunotoxins (Thorpe *et al.*, 1985), may have better *in vivo* stability because these RIPs are positively charged at neutral pH and, as hypothesised for saporin immunotoxins, they may interact non-covalently with the negatively charged antibody molecule to shield the disulphide bond from cleavage by thiol-containing compounds or proteins in the animal.

The work performed in Bologna was supported by a contract from the Consiglio Nazionale delle Ricerche, Rome, by grants from the Associazione Italiana per la Ricerca sul Cancro, Milan, and from the Ministero della Pubblica Istruzione, Rome and by the Pallotti's Legacy for Cancer Research. We thank Mrs A. Becket for typing the manuscript.

### References

BARBIERI, L. & STIRPE, F. (1982). Ribosome-inactivating proteins from plants: Properties and possible uses. *Cancer Surveys*, **1**, 489.

BARBIERI, L., ZAMBONI, M., LORENZONI, E., MONTANARO, L., SPERTI, S. & STIRPE, F. (1980). Inhibition of protein synthesis *in vitro* by proteins from the seeds of *Momordica charantia* (bitter pear melon). *Biochem. J.*, **186**, 443.

BLAKEY, D.C. & THORPE, P.E. (1988). The prevention of carbohydrate-mediated clearance of ricin-containing immunotoxins by the liver. In *Immunotoxins*, Frankel, A. (ed) p. 457. Martinus Nijhoff Publishers: Boston.

BLAKEY, D.C., WATSON, G.J., KNOWLES, P.P. & THORPE, P.E. (1987*a*). Effect of chemical deglycosylation of ricin A-chain on the *in vivo* fate and cytotoxic activity of an immunotoxin composed of ricin A-chain and anti-Thy 1.1 antibody. *Cancer Res.* **47**, 947.

BLAKEY, D.C., WAWRZYNCZAK, E.J., STIRPE, F. & THORPE, P.E. (1987*b*). Anti-tumour activity of a panel of anti-Thy 1.1 immunotoxins made with different ribosome-inactivating proteins. In *Membrane-Mediated Cytotoxicity*, Bonavida, B. & Collier, R.J. (eds) p. 195. Alan R. Liss: New York.

BLYTHMAN, H.E., CASELLAS, P., GROS, O. & 5 others (1981). Immunotoxins: Hybrid molecules of monoclonal antibodies and a toxin subunit specifically kill tumour cells. *Nature*, **290**, 145.

COLOMBATTI, M., NABHOLZ, M., GROS, O. & BRON, C. (1983). Selective killing of target cells by antibody-ricin A chain or antibody-gelonin hybrid molecules: Comparison of cytotoxic potency and use in immunoselection procedures. *J. Immunol.*, **131**, 3091.

FALASCA, A., GASPERI-CAMPANI, A., ABBONDANZA, A., BARBIERI, L. & STIRPE, F. (1982). Properties of the ribosome-inactivating proteins gelonin, *Momordica charantia* inhibitor, and dianthins. *Biochem. J.*, **207**, 505.

GLENNIE, M.J., McBRIDE, H.M., STIRPE, F., THORPE, P.E., WORTH, A.T. & STEVENSON, G.T. (1987). Emergence of immunoglobulin variants following treatment of a B cell leukemia with an immunotoxin composed of antiidiotypic antibody and saporin. *J. Exp. Med.*, **166**, 43.

LAMBERT, J.M., SENTER, P.D., YAU-YOUNG, A., BLATTLER, W.A. & GOLDMACHER, V.S. (1985). Purified immunotoxins that are reactive with human lymphoid cells. *J. Biol. Chem.*, **260**, 12035.

LETVIN, N.L., GOLDMACHER, V.S., RITZ, J., YETZ, J.M., SCHLOSSMAN, S.F. & LAMBERT, J.M. (1986). *In vivo* administration of lymphocyte-specific monoclonal antibodies in nonhuman primates. *J. Clin. Invest.*, **77**, 977.

MASON, D.W. & WILLIAMS, A.F. (1980). The kinetics of antibody binding to membrane antigens in solution and at the cell surface. *Biochem. J.*, **187**, 1.

MASUHO, M., KISHIDA, K. & HARA, T. (1982). Targeting of the antiviral protein from *Phytolacca americana* with an antibody. *Biochem. Biophys. Res. Commun.*, **105**, 462.

RAMAKRISHNAN, S. & HOUSTON, L.L. (1984*a*). Comparison of the selective cytotoxic effects of immunotoxins containing ricin A chain or pokeweed antiviral protein and anti-Thy 1.1 monoclonal antibodies. *Cancer Res.*, **44**, 201.

RAMAKRISHNAN, S. & HOUSTON, L.L. (1984*b*). Prevention of growth of leukemia cells in mice by monoclonal antibodies directed against Thy 1.1 antigen disulfide linked to two ribosomal inhibitors: Pokeweed antiviral protein or ricin A chain. *Cancer Res.*, **44**, 1398.

RAMAKRISHNAN, S. & HOUSTON, L.L. (1985). Immunological and biological stability of immunotoxins *in vivo* as studied by the clearance of disulfide-linked pokeweed antiviral protein-antibody conjugates from blood. *Cancer Res.*, **45**, 2031.

SCOTT, C.F., Jr., GOLDMACHER, V.S., LAMBERT, J.M., JACKSON, J.V. & McINTYRE, G.D. (1987*a*). An immunotoxin composed of a monoclonal antitransferrin receptor antibody linked by a disulfide bond to the ribosome-inactivating protein gelonin: Potent *in vitro* and *in vivo* effects against human tumors. *J. Natl Cancer Inst.*, **79**, 1163.

SCOTT, C.F., Jr., LAMBERT, J.M., GOLDMACHER, V.S. & 4 others (1987*b*). The pharmacokinetics and toxicity of murine monoclonal antibodies and of gelonin conjugates of these antibodies. *Int. J. Immunopharmac.*, **9**, 211.

SIVAM, G., PEARSON, J.W., BOHN, W., OLDHAM, R.K., SADOFF, J.C. & MORGAN, A.C. Jr. (1987). Immunotoxins to a human melanoma-associated antigen: Comparison of gelonin with ricin and other A chain conjugates. *Cancer Res.*, **47**, 3169.

STIRPE, F., BAILEY, S., MILLER, S.P. & BODLEY, J.W. (1988). Modification of ribosomal RNA by ribosome-inactivating proteins from plants. *Nucl. Acids Res.*, **16**, 1349.

STIRPE, F. & BARBIERI, L. (1986). Ribosome-inactivating proteins up to date. *FEBS Lett.*, **195**, 1.

STIRPE, F., BARBIERI, L., BATTELLI, M.G. & 4 others (1986). Bryodin, a ribosome-inactivating protein from the roots of *Bryonia dioica* L. (white bryony). *Biochem. J.*, **240**, 659.

THORPE, P.E., BLAKEY, D.C., BROWN, A.N.F. & 5 others (1987). Comparison of two anti-Thy 1.1-abrin A chain immunotoxins prepared with different cross-linking agents: Antitumor effects, *in vivo* fate, and tumor cell mutants. *J. Natl Cancer Inst.*, **79**, 1101.

THORPE, P.E., BROWN, A.N.F., BREMNER, J.A.G., Jr., FOXWELL, B.M.J. & STIRPE, F. (1985). An immunotoxin composed of monoclonal anti-Thy 1.1 antibody and a ribosome-inactivating protein from *Saponaria officinalis*: Potent antitumor effects *in vitro* and *in vivo*. *J. Natl Cancer Inst.*, **75**, 151.

THORPE, P.E., BROWN, A.N.F., ROSS, W.C.J. & 5 others (1981). Cytotoxicity acquired by conjugation of an anti-Thy 1.1 monoclonal antibody and the ribosome-inactivating protein, gelonin. *Eur. J. Biochem.*, **116**, 447.

UCKUN, F.M., RAMAKRISHNAN, S. & HOUSTON, L.L. (1985). Increased efficiency in selective elimination of leukemia cells by a combination of a stable derivative of cyclophosphamide and a human B-cell specific immunotoxin containing pokeweed antiviral protein. *Cancer Res.*, **45**, 69.

WAWRZYNCZAK, E.J. & THORPE, P.E. (1988). Effect of chemical linkage upon the stability and cytotoxic activity of A chain immunotoxins. In *Immunotoxins*, Frankel, A. (ed) p. 239, Martinus Nijhoff Publishers: Boston.

WIELS, J., JUNQUA, S., DUJARDIN, P., le PECQ, J.B. & TURSZ, T. (1984). Properties of immunotoxins against a glycolipid antigen associated with Burkitt's lymphoma. *Cancer Res.*, **44**, 129.